# Crystal structure of bis­[tetra­kis­(tri­phenyl­phosphane-κ*P*)silver(I)] (nitrilo­tri­acetato-κ^4^ *N*,*O*,*O*′,*O*′′)(tri­phenyl­phosphane-κ*P*)argentate(I) with an unknown amount of methanol as solvate

**DOI:** 10.1107/S2056989016001262

**Published:** 2016-02-10

**Authors:** Julian Noll, Marcus Korb, Heinrich Lang

**Affiliations:** a Technische Universität Chemnitz, Fakultät für Naturwissenschaften, Institut für Chemie, Anorganische Chemie, D-09107 Chemnitz, Germany

**Keywords:** crystal structure, silver, triphenyl phosphine, non-coordinating anion, SQUEEZE

## Abstract

The structure of the title compound, [Ag(C_18_H_15_P)_4_]_2_[Ag(C_6_H_6_NO_6_)(C_18_H_15_P)], exhibits trigonal (*P*-3) symmetry, with a *C*_3_ axis through all three complex ions, resulting in an asymmetric unit that contains one third of the atoms present in the formula unit. The formula unit thus contains two of the cations, one anion and disordered mol­ecules of methanol as the packing solvent. Attempts to refine the solvent model were unsuccessful, indicating uninter­pretable disorder. Thus, the SQUEEZE procedure in *PLATON* [Spek (2015 ▸). *Acta Cryst*. C**71**, 9–18] was applied, accounting for 670 electrons per unit cell, representing approximately 18 mol­ecules of methanol in the formula unit. The stated crystal data for *M*_r_, μ *etc* do not take these into account.

## Abstract 2

The structure of the title compound exhibits a trigonal (*P*-3) symmetry, with a *C*_3_ axis through all three complex ions, resulting in an asymmetric unit that contains one third of the atoms present in the formula unit. Attempts to refine the solvent model were unsuccessful, indicating uninter­pretable disorder, which was handled using SQUEEZE.

## Chemical context   

Metal nanoparticles are well known in the literature for their use in various applications, *e.g*., in joining processes (Hausner *et al.*, 2014 ▸), catalysis (Steffan *et al.*, 2009 ▸; Zhang *et al.*, 2015 ▸) and electronics (Gilles *et al.*, 2013 ▸; Scheideler *et al.*, 2015 ▸). This is caused by the size and shape-dependent properties of the nanoparticles (Wilcoxon & Abrams, 2006 ▸). The formation of nanoparticles requires a metal source, reducing as well as stabilizing agents, and can be achieved by the decomposition of precursors either by heat (Adner *et al.*, 2013 ▸) or light (Schliebe *et al.*, 2013 ▸). However, to combine the metal source and reducing agents in one mol­ecule, silver (I) carboxyl­ates are convenient compounds. They are known for their light sensitivity and their ability to decompose thermally into elemental silver (Fields & Meyerson, 1976 ▸), but due to their low solubility, the corresponding phosphine complexes can also be used. In the context of this approach, the title compound [Ag(C_18_H_15_P)_4_]_2_[Ag(C_6_H_6_NO_6_)(C_18_H_15_P)], (I), was obtained as a methanol solvate of unknown composition by the reaction of the tri-silver salt of nitrilo­tri­acetic acid with tri­phenyl­phosphane.
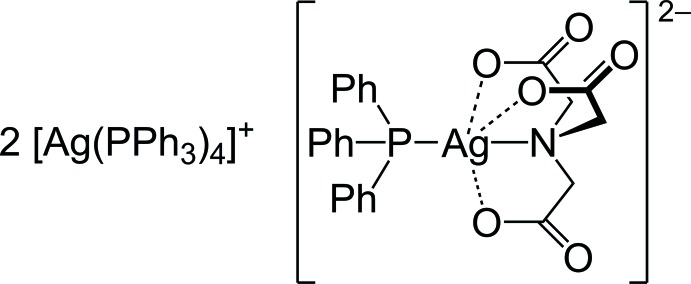


## Structural commentary   

**Figure 1:**
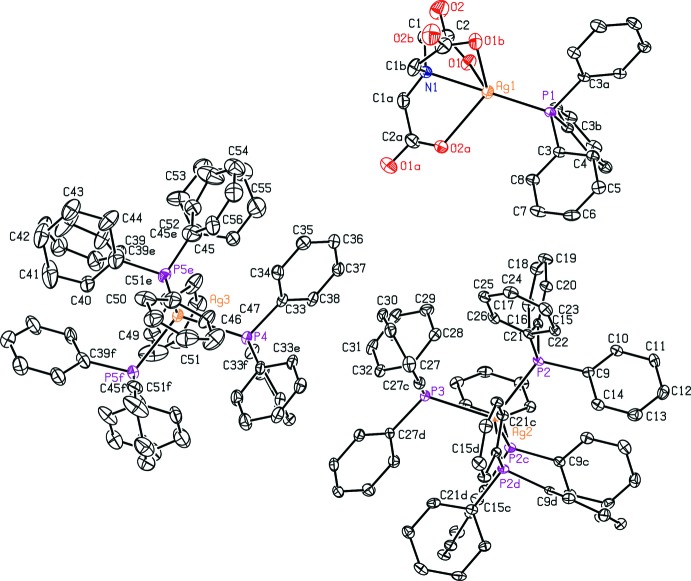
The structures of the molecular components of (I), with displacement ellipsoids drawn at the 50% probability level. All H atoms have been omitted for clarity. [Symmetry codes: (*a*) −*x* + *y* + 1, −*x* + 2, *z*; (*b*) −*y* + 2, *x* − *y* + 1, *z*; (*c*/*f*) −*x* + *y* + 1, −*x* + 1, *z*; (*d*/*e*) −*y* + 1, *x* − *y*, *z*.]

The asymmetric unit of the title compound presents one-third of the formula unit (Fig. 1 ▸), which contains two of the cations, one anion and approximately 18 mol­ecules of methanol. The whole compound can thus be generated using the *C*_3_ symmetry operations (Fig. 1 ▸) present for each ion. Thus, the tetra­kis­(tri­phenyl­phosphino)silver cations are built up by one PPh_3_ ligand, the silver ion and one P(Ph)_1_ fragment in the asymmetric unit (Fig. 1 ▸; *c*/*f*, −*x* + *y* + 1, −*x* + 1, *z*; *d*/*e*, −*y* + 1, *x* − *y*, *z*). A tetra­hedral coordination environment [P—Ag—P = 108.82 (3)–110.11 (3)°] is observed for the silver ions of the cationic fragments with anti-periplanar torsion angles [P—Ag—P—C 175.35 (15) and 177.9 (3)°] between the phenyl rings of the PPh_3_ ligand towards the opposite Ag—P bond.

With regard to the anionic silver-NTA (NTA = nitrilo­tri­acetate) complex, only one acetato ligand, atoms N1 and Ag1, and a P(Ph)_1_ fragment are present in the asymmetric unit. In the whole *C_3_*-symmetric anion [symmetry codes: (*a*) −*x* + *y* + 1, −*x* + 2, *z*; (*b*) −*y* + 2, *x* − *y* + 1, *z*; Fig. 1 ▸], the silver ion is coordinated by one PPh_3_ ligand and the N1 atom of the NTA mol­ecule, with a linear N1—Ag1—P1 environment (180.0°). However, a further inter­action between one oxygen atom of each carboxyl­ato moiety and a silver atom within the range of the van der Waals radii [2.599 (4) Å, Σ = 3.24 Å] (Spek, 2009 ▸) is present, resulting in a strongly distorted trigonal–bipyramidal complex geometry. The acetato moieties are rotated in a staggered fashion towards the phenyl rings of the PPh_3_ ligand with *X*—Ag1—P1—C3 torsion angles of 70.1 (3)° (*X* = C1) and 30.59 (18)° (*X* = O1).

**Figure 2:**
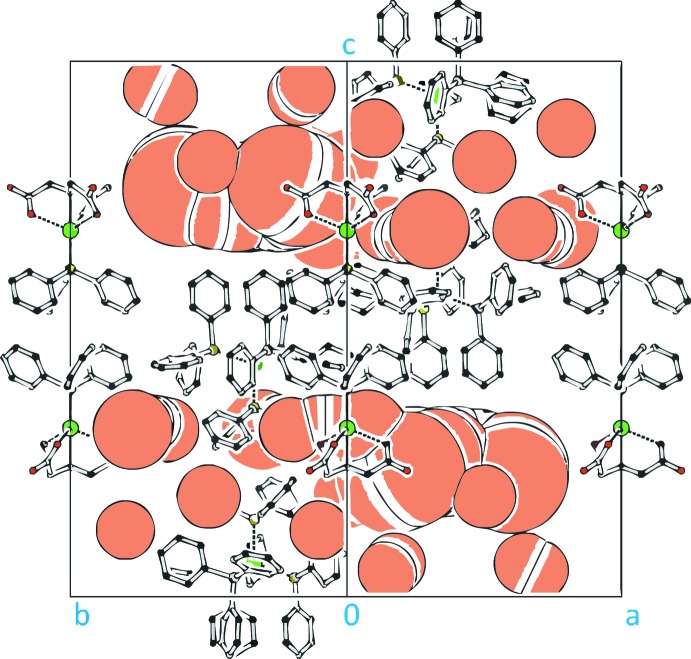
*PLUTON* cavity plot of the crystal packing of (I) in a view along [110] showing the cavities (pale red) occupied by the disordered methanol solvent. All H atoms have been omitted for clarity.

The unit cell contains approximately 36 extensively disordered mol­ecules of methanol (*i.e*., six mol­ecules of MeOH in the asymmetric unit) that were accounted for using the SQUEEZE routine in *PLATON* (Spek, 2015 ▸) (Fig. 2 ▸, see also: *Refinement*).

## Supra­molecular features   

**Figure 3:**
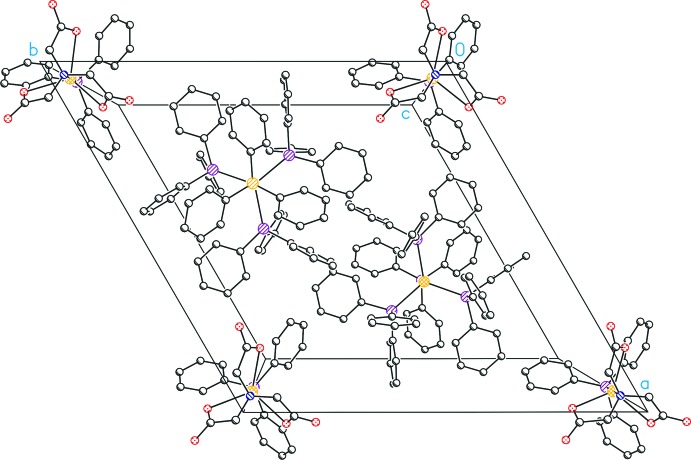
Crystal packing of the mol­ecular structure of (I) with the view along [001]. All H atoms have been omitted for clarity.

The anions of (I) are packed along the *c* axis through the N—Ag—P bond (Figs. 2 ▸ and 3 ▸) with the PPh_3_ ligands of two ions facing each other. The cations, placed within the cell (Fig. 3 ▸) form a layer type structure parallel to (001) (Fig. 2 ▸), whereas the anions are placed on the cell axes. The omitted methanol solvent is packed above and below these (001) planes, indicating the potential presence of hydrogen bridge-bonds to the carboxyl­ato-oxygen atoms (Fig. 2 ▸). Inter- or intra­molecular π inter­actions are not present.

## Database survey   

Since the first synthesis of nitrilo­tri­acetic acid (Polstorff & Meyer, 1912 ▸), a wide diversity of complexes with this mol­ecule containing several metals have been synthesized over the last few decades (Hoard *et al.*, 1968 ▸; Dung *et al.*, 1988 ▸; Kumari *et al.*, 2012 ▸). In contrast, only three crystal structures in which the N atom of nitrilo­tri­acetic acid is bonded to silver(I) are known (Sun *et al.*, 2011 ▸; Chen *et al.*, 2005 ▸), whereas coordin­ation of the O atom of nitrilo­tri­acetic acid to silver(I) is more common (Novitchi *et al.*, 2010 ▸; Sun *et al.*, 2011 ▸; Chen *et al.*, 2005 ▸; Liang *et al.*, 1964 ▸). However, many silver(I) complexes with phosphanes as ligands are known in the literature (Frenzel *et al.*, 2014 ▸; Rüffer *et al.*, 2011 ▸; Jakob *et al.*, 2005 ▸). Likewise, the coordination of four tri­phenyl­phosphane ligands to one silver(I) ion has occurred in a variety of possible structural motifs in the last few decades (Pelizzi *et al.*, 1984 ▸; Ng, 2012 ▸; Bowmaker *et al.*, 1990 ▸).

## Synthesis and crystallization   

**Synthesis of trisilvernitrilo­tri­acetate:**

Colorless [(AgO_2_CCH_2_)_3_N] was prepared by an alternative route to the synthetic methodologies reported by Cotrait and Joussot-Dubien (1966 ▸), *i.e*., by the reaction of nitrilo­tri­acetic acid tris­odium salt with [AgNO_3_] in water at ambient temperature, and with exclusion of light (Noll *et al.*, 2014 ▸). It is advisable to consecutively wash the respective silver carboxyl­ate with water and diethyl ether to obtain a pure product.

**Synthesis of bis­[tetra­kis­(tri­phenyl­phosphane-κ** ***P*** **)silver(I)] (nitrilo­tri­acetato-κ^4^** ***N***,***O***,***O*** **′**,***O*** **′′)(tri­phenyl­phosphane-κ** ***P*** **)argen­tate(I) methanol solvate (I):**

For this reaction, tri­phenyl­phosphane (0.385 g, 1,47 mmol, 3 eq) was diluted in 30 mL of ethanol and 1 equiv. (0.25 g, 0,49 mmol) of tri-silver-nitrilo­tri­acetate suspended in 30 mL of ethanol was added dropwise. After stirring for 12 h in the dark, the solution was filtered and the solvent removed *in vacuo*. Suitable crystals were obtained by diffusion of hexane into a methanol solution containing (I) at ambient temperature.

M.p. 390 K. ^1^H NMR (CD_3_OD, p.p.m.) δ: 3.72 (*s*, 6 H), 7.08–7.12 (*m*, CH^*o*^Ph, 54 H), 7.14–7.17 (*m*, CH^*m*^Ph, 54 H), 7.39–7.43 (*m*, CH^*p*^Ph, 27 H). ^13^C {^1^H} (CD_3_OD, p.p.m.) δ: 58.35 (*s*, CH_2_) 130.26 (*d*, C^*m*^Ph, ^3^ *J*_CP_ = 9.36 Hz), 131.83 (*d*, C^*p*^Ph, ^4^ *J*_CP_ = 1.17 Hz), 132.95 (*d*, C^*i*^Ph, ^1^ *J*_CP_ = 24.54 Hz), 134.88 (*d*, C^*o*^Ph, ^2^ *J*_CP_ = 15.72 Hz). ^31^P {^1^H} (CD_3_OD, p.p.m.) δ: 6.82. IR (KBr, cm^−1^): = 3417 (*b*), 3053 (*s*), 1890 (*w*), 1636 (*b*), 1478 (*m*), 743 (*s*), 697 (*s*).

All reagents and solvents were obtained commercially and used without further purification.

## Refinement   

Crystal data, data collection and structure refinement details are summarized in Table 1 ▸. C-bonded H atoms were placed in calculated positions and constrained to ride on their parent atoms with *U*_iso_(H) = 1.2*U*_eq_(C) and a C—H distance of 0.93 Å for aromatic and 0.97 Å for methyl­ene H atoms. Attempts to avoid the differences in the anisotropic displacement parameters (Hirshfeld, 1976 ▸) of P5 and C45 by using RIGU, SIMU/ISOR, or EADP instructions were not successful (McArdle, 1995 ▸; Sheldrick, 2008 ▸).

The crystal contains disordered methanol mol­ecules as the packing solvent. Attempts to refine an adequate disordered solvent model failed, presumably due to the large number of mol­ecules involved and the restraints required for an anisotropic refinement. Thus, the SQUEEZE procedure (Spek, 2015 ▸) of *PLATON* (Spek 2003 ▸, 2009 ▸) was used to delete the solvent contribution. This treatment decreased the *R*_1_ value from 0.0920 to 0.0664 and the *wR*_2_ value from 0.2832 to 0.1849 by excluding a volume of 4050.5 Å^3^ (40.5% of the total cell volume) and 670 electrons, respectively. The excluded volume is shown in Fig. 2 ▸ represented by a *PLATON* cavity plot (Spek 2003 ▸, 2009 ▸) with the spheres representing the cavities that are filled with the disordered solvent. Given the number of electrons excluded by the SQUEEZE procedure, an estimate of about 36 methanol mol­ecules can be calculated for the whole unit cell, which corresponds to approximately six methanol mol­ecules per asymmetric unit. The stated crystal data for *M*_r_, μ *etc* (Table 1 ▸) do not take these into account.

## Supplementary Material

Crystal structure: contains datablock(s) I. DOI: 10.1107/S2056989016001262/pk2571sup1.cif

Structure factors: contains datablock(s) I. DOI: 10.1107/S2056989016001262/pk2571Isup2.hkl

CCDC reference: 1448527

Additional supporting information:  crystallographic information; 3D view; checkCIF report

## Figures and Tables

**Table 1:** Experimental details

Crystal data	
Chemical formula	[Ag(C_18_H_15_P)_4_]_2_[Ag(C_6_H_6_NO_6_)(C_18_H_15_P)]
*M*_r_	2872.15
Crystal system, space group	Trigonal, *P* 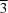
Temperature (K)	110
*a*, *c* (Å)	19.0095 (5), 31.9862 (10)
*V* (Å^3^)	10010.0 (6)
*Z*	2
Radiation type	Mo *K*α
μ (mm^−1^)	0.40
Crystal size (mm)	0.2 × 0.2 × 0.2

Data collection	
Diffractometer	Oxford Gemini S
Absorption correction	Multi-scan (*CrysAlis RED*; Oxford Diffraction, 2006 ▸)
*T*_min_, *T*_max_	0.699, 1.000
No. of measured, independent and observed [*I* > 2σ(*I*)] reflections	32447, 12365, 8561
*R*_int_	0.049
(sin θ/λ)_max_ (Å^−1^)	0.606

Refinement	
*R*[*F* ^2^ > 2σ(*F* ^2^)], *wR*(*F* ^2^), *S*	0.066, 0.197, 1.05
No. of reflections	12365
No. of parameters	572
H-atom treatment	H-atom parameters constrained
Δρ_max_, Δρ_min_ (e Å^−3^)	1.34, −0.64

Computer programs: *CrysAlis CCD* and *CrysAlis RED* (Oxford Diffraction, 2006 ▸), *SHELXS97* and *SHELXTL* (Sheldrick, 2008 ▸), *SHELXL2013* (Sheldrick, 2015 ▸), *ORTEP-3 for Windows* and *WinGX* (Farrugia, 2012 ▸) and *publCIF* (Westrip, 2010 ▸).

## supplementary crystallographic information

### Crystal data

**Table:**

[Ag(C_18_H_15_P)_4_]_2_[Ag(C_6_H_6_NO_6_)(C_18_H_15_P)]	*D*_x_ = 0.953 Mg m^−^^3^
*M**_r_* = 2872.15	Mo *K*α radiation, λ = 0.71073 Å
Trigonal, *P*3	Cell parameters from 6868 reflections
*a* = 19.0095 (5) Å	θ = 3.3–27.6°
*c* = 31.9862 (10) Å	µ = 0.40 mm^−^^1^
*V* = 10010.0 (6) Å^3^	*T* = 110 K
*Z* = 2	Block, colorless
*F*(000) = 2960	0.2 × 0.2 × 0.2 mm

### Data collection

**Table:**

Oxford Gemini S diffractometer	*R*_int_ = 0.049
ω scans	θ_max_ = 25.5°, θ_min_ = 3.2°
Absorption correction: multi-scan (*CrysAlis RED*; Oxford Diffraction, 2006)	*h* = −17→22
*T*_min_ = 0.699, *T*_max_ = 1.000	*k* = −18→23
32447 measured reflections	*l* = −38→24
12365 independent reflections	2 standard reflections every 50 reflections
8561 reflections with *I* > 2σ(*I*)	intensity decay: none

### Refinement

**Table:**

Refinement on *F*^2^	0 restraints
Least-squares matrix: full	Hydrogen site location: inferred from neighbouring sites
*R*[*F*^2^ > 2σ(*F*^2^)] = 0.066	H-atom parameters constrained
*wR*(*F*^2^) = 0.197	*w* = 1/[σ^2^(*F*_o_^2^) + (0.101*P*)^2^ + 10.4365*P*] where *P* = (*F*_o_^2^ + 2*F*_c_^2^)/3
*S* = 1.05	(Δ/σ)_max_ = 0.001
12365 reflections	Δρ_max_ = 1.34 e Å^−^^3^
572 parameters	Δρ_min_ = −0.64 e Å^−^^3^

### Special details

**Table:**

Geometry. All e.s.d.'s (except the e.s.d. in the dihedral angle between two l.s. planes) are estimated using the full covariance matrix. The cell e.s.d.'s are taken into account individually in the estimation of e.s.d.'s in distances, angles and torsion angles; correlations between e.s.d.'s in cell parameters are only used when they are defined by crystal symmetry. An approximate (isotropic) treatment of cell e.s.d.'s is used for estimating e.s.d.'s involving l.s. planes.
Refinement. Refinement of **F**^2^ against ALL reflections. The weighted **R**-factor *wR* and goodness of fit **S** are based on **F**^2^, conventional **R**-factors **R** are based on **F**, with **F** set to zero for negative **F**^2^. The threshold expression of **F**^2^ > σ(**F**^2^) is used only for calculating **R**-factors(gt) *etc*. and is not relevant to the choice of reflections for refinement. **R**-factors based on **F**^2^ are statistically about twice as large as those based on **F**, and **R**-factors based on ALL data will be even larger.

### Fractional atomic coordinates and isotropic or equivalent isotropic displacement parameters (Å^2^)

**Table:**

	*x*	*y*	*z*	*U*_iso_*/*U*_eq_	
C1	0.9981 (4)	1.0723 (4)	0.77087 (17)	0.0510 (15)	
H1A	1.0514	1.1195	0.7667	0.061*	
H1B	0.9864	1.0679	0.8006	0.061*	
C2	0.9347 (4)	1.0855 (4)	0.7476 (2)	0.0520 (15)	
C3	1.0199 (3)	0.9244 (3)	0.58793 (14)	0.0249 (10)	
C4	1.0648 (3)	0.9386 (3)	0.55126 (15)	0.0290 (11)	
H4	1.0874	0.9891	0.5382	0.035*	
C5	1.0757 (3)	0.8780 (3)	0.53427 (16)	0.0341 (12)	
H5	1.1063	0.8879	0.5100	0.041*	
C6	1.0413 (3)	0.8024 (3)	0.55318 (17)	0.0378 (13)	
H6	1.0474	0.7611	0.5412	0.045*	
C7	0.9980 (3)	0.7884 (3)	0.58994 (18)	0.0402 (13)	
H7	0.9758	0.7382	0.6031	0.048*	
C8	0.9878 (3)	0.8493 (3)	0.60683 (16)	0.0317 (11)	
H8	0.9586	0.8397	0.6315	0.038*	
C9	0.7784 (2)	0.4908 (3)	0.48160 (13)	0.0227 (9)	
C10	0.7818 (3)	0.5545 (3)	0.45909 (15)	0.0278 (10)	
H10	0.7772	0.5951	0.4730	0.033*	
C11	0.7920 (3)	0.5586 (3)	0.41567 (15)	0.0330 (11)	
H11	0.7935	0.6013	0.4007	0.040*	
C12	0.7997 (3)	0.4996 (3)	0.39533 (15)	0.0362 (12)	
H12	0.8076	0.5027	0.3665	0.043*	
C13	0.7958 (3)	0.4347 (3)	0.41765 (17)	0.0395 (13)	
H13	0.8003	0.3942	0.4037	0.047*	
C14	0.7853 (3)	0.4306 (3)	0.46039 (15)	0.0304 (11)	
H14	0.7828	0.3873	0.4752	0.036*	
C15	0.7362 (3)	0.5570 (3)	0.55164 (13)	0.0218 (9)	
C16	0.6549 (3)	0.5326 (3)	0.55782 (14)	0.0257 (10)	
H16	0.6157	0.4781	0.5551	0.031*	
C17	0.6318 (3)	0.5894 (3)	0.56809 (14)	0.0319 (11)	
H17	0.5772	0.5723	0.5725	0.038*	
C18	0.6885 (3)	0.6700 (3)	0.57182 (15)	0.0345 (12)	
H18	0.6725	0.7076	0.5783	0.041*	
C19	0.7696 (3)	0.6952 (3)	0.56595 (16)	0.0372 (12)	
H19	0.8081	0.7499	0.5688	0.045*	
C20	0.7945 (3)	0.6395 (3)	0.55582 (14)	0.0284 (10)	
H20	0.8492	0.6569	0.5519	0.034*	
C21	0.8692 (3)	0.5295 (2)	0.55785 (14)	0.0216 (9)	
C22	0.9362 (3)	0.5552 (3)	0.53165 (15)	0.0275 (10)	
H22	0.9289	0.5481	0.5029	0.033*	
C23	1.0147 (3)	0.5917 (3)	0.54861 (16)	0.0348 (12)	
H23	1.0594	0.6084	0.5312	0.042*	
C24	1.0255 (3)	0.6028 (3)	0.59136 (16)	0.0350 (12)	
H24	1.0775	0.6275	0.6027	0.042*	
C25	0.9584 (3)	0.5770 (3)	0.61745 (16)	0.0324 (11)	
H25	0.9655	0.5848	0.6462	0.039*	
C26	0.8818 (3)	0.5399 (3)	0.60066 (15)	0.0286 (10)	
H26	0.8373	0.5213	0.6184	0.034*	
C27	0.7674 (3)	0.3799 (3)	0.67114 (13)	0.0263 (10)	
C28	0.7918 (3)	0.4372 (3)	0.70267 (14)	0.0293 (11)	
H28	0.7561	0.4534	0.7126	0.035*	
C29	0.8694 (3)	0.4708 (3)	0.71949 (14)	0.0367 (12)	
H29	0.8862	0.5106	0.7400	0.044*	
C30	0.9217 (3)	0.4449 (3)	0.70572 (15)	0.0388 (13)	
H30	0.9729	0.4661	0.7177	0.047*	
C31	0.8982 (3)	0.3876 (3)	0.67416 (16)	0.0392 (13)	
H31	0.9336	0.3707	0.6647	0.047*	
C32	0.8219 (3)	0.3560 (3)	0.65699 (15)	0.0323 (11)	
H32	0.8062	0.3180	0.6356	0.039*	
C33	0.7197 (3)	0.4342 (3)	0.83069 (14)	0.0324 (11)	
C34	0.7969 (4)	0.4884 (3)	0.84516 (16)	0.0457 (14)	
H34	0.8199	0.4729	0.8663	0.055*	
C35	0.8402 (4)	0.5664 (4)	0.82803 (17)	0.0517 (16)	
H35	0.8922	0.6027	0.8376	0.062*	
C36	0.8046 (4)	0.5899 (4)	0.79617 (16)	0.0465 (14)	
H36	0.8325	0.6419	0.7849	0.056*	
C37	0.7297 (3)	0.5358 (3)	0.78221 (16)	0.0390 (13)	
H37	0.7064	0.5510	0.7611	0.047*	
C38	0.6863 (3)	0.4572 (3)	0.79896 (14)	0.0372 (12)	
H38	0.6350	0.4206	0.7887	0.045*	
C39	0.8225 (4)	0.4078 (5)	1.0209 (2)	0.073 (2)	
C40	0.7801 (4)	0.3373 (4)	1.04272 (19)	0.067 (2)	
H40	0.7470	0.2900	1.0279	0.080*	
C41	0.7834 (5)	0.3322 (7)	1.0857 (3)	0.126 (5)	
H41	0.7567	0.2825	1.0995	0.152*	
C42	0.8294 (5)	0.4061 (6)	1.1079 (2)	0.088 (3)	
H42	0.8292	0.4063	1.1369	0.106*	
C43	0.8754 (5)	0.4791 (6)	1.0857 (2)	0.080 (2)	
H43	0.9086	0.5266	1.1004	0.096*	
C44	0.8725 (4)	0.4820 (5)	1.0421 (2)	0.077 (2)	
H44	0.9021	0.5306	1.0276	0.092*	
C45	0.8742 (4)	0.3609 (4)	0.9471 (2)	0.0607 (18)	
C46	0.8732 (4)	0.3449 (4)	0.90415 (18)	0.0551 (17)	
H46	0.8444	0.3588	0.8856	0.066*	
C47	0.9159 (5)	0.3081 (6)	0.8899 (3)	0.090 (3)	
H47	0.9131	0.2939	0.8619	0.108*	
C48	0.9638 (5)	0.2919 (5)	0.9180 (3)	0.084 (2)	
H48	0.9943	0.2693	0.9084	0.101*	
C49	0.9645 (4)	0.3106 (4)	0.9606 (2)	0.071 (2)	
H49	0.9952	0.2992	0.9791	0.086*	
C50	0.9200 (4)	0.3458 (4)	0.9760 (3)	0.069 (2)	
H50	0.9211	0.3585	1.0042	0.083*	
C51	0.8718 (4)	0.5109 (4)	0.9494 (2)	0.0674 (19)	
C52	0.8434 (3)	0.5648 (4)	0.95708 (19)	0.0499 (15)	
H52	0.7919	0.5433	0.9688	0.060*	
C53	0.8828 (5)	0.6430 (5)	0.9493 (3)	0.104 (3)	
H53	0.8630	0.6765	0.9578	0.124*	
C54	0.9596 (5)	0.6749 (5)	0.9266 (3)	0.082 (2)	
H54	0.9871	0.7285	0.9173	0.098*	
C55	0.9905 (5)	0.6236 (5)	0.9190 (2)	0.074 (2)	
H55	1.0408	0.6451	0.9059	0.088*	
C56	0.9503 (4)	0.5414 (5)	0.93011 (19)	0.067 (2)	
H56	0.9728	0.5085	0.9253	0.080*	
O1	0.9044 (2)	1.0481 (2)	0.71512 (12)	0.0467 (10)	
O2	0.9195 (3)	1.1367 (3)	0.76428 (15)	0.0755 (14)	
P1	1.0000	1.0000	0.61143 (6)	0.0240 (4)	
P2	0.76469 (7)	0.48109 (7)	0.53863 (4)	0.0214 (3)	
P3	0.6667	0.3333	0.64650 (6)	0.0230 (4)	
P4	0.6667	0.3333	0.85547 (7)	0.0315 (5)	
P5	0.81418 (9)	0.40685 (9)	0.96398 (4)	0.0393 (3)	
Ag1	1.0000	1.0000	0.68457 (2)	0.03511 (19)	
Ag2	0.6667	0.3333	0.56556 (2)	0.01929 (15)	
Ag3	0.6667	0.3333	0.93618 (2)	0.03244 (18)	
N1	1.0000	1.0000	0.7572 (2)	0.0305 (16)	

### Atomic displacement parameters (Å^2^)

**Table:**

	*U*^11^	*U*^22^	*U*^33^	*U*^12^	*U*^13^	*U*^23^
C1	0.074 (4)	0.047 (3)	0.034 (3)	0.032 (3)	0.005 (3)	−0.003 (3)
C2	0.061 (4)	0.053 (4)	0.055 (4)	0.038 (3)	0.007 (3)	−0.002 (3)
C3	0.020 (2)	0.022 (2)	0.034 (3)	0.012 (2)	−0.001 (2)	−0.001 (2)
C4	0.028 (3)	0.026 (2)	0.037 (3)	0.016 (2)	−0.003 (2)	0.001 (2)
C5	0.030 (3)	0.037 (3)	0.040 (3)	0.020 (2)	−0.001 (2)	−0.002 (2)
C6	0.030 (3)	0.029 (3)	0.058 (3)	0.017 (2)	−0.004 (3)	−0.013 (2)
C7	0.034 (3)	0.022 (3)	0.062 (4)	0.012 (2)	0.002 (3)	−0.001 (2)
C8	0.028 (3)	0.028 (3)	0.040 (3)	0.015 (2)	0.002 (2)	−0.001 (2)
C9	0.013 (2)	0.024 (2)	0.029 (2)	0.0080 (19)	−0.0010 (18)	−0.0022 (19)
C10	0.025 (2)	0.023 (2)	0.035 (3)	0.012 (2)	0.003 (2)	0.002 (2)
C11	0.035 (3)	0.036 (3)	0.031 (3)	0.019 (2)	0.001 (2)	0.011 (2)
C12	0.030 (3)	0.048 (3)	0.027 (3)	0.016 (2)	0.003 (2)	−0.001 (2)
C13	0.038 (3)	0.035 (3)	0.045 (3)	0.018 (3)	0.005 (2)	−0.007 (2)
C14	0.027 (3)	0.025 (2)	0.038 (3)	0.012 (2)	0.003 (2)	0.003 (2)
C15	0.025 (2)	0.022 (2)	0.021 (2)	0.013 (2)	0.0014 (18)	0.0025 (18)
C16	0.023 (2)	0.023 (2)	0.031 (2)	0.012 (2)	0.000 (2)	0.0026 (19)
C17	0.024 (3)	0.044 (3)	0.034 (3)	0.022 (2)	0.001 (2)	0.002 (2)
C18	0.042 (3)	0.036 (3)	0.038 (3)	0.029 (3)	0.000 (2)	−0.002 (2)
C19	0.039 (3)	0.022 (3)	0.048 (3)	0.014 (2)	−0.002 (2)	0.000 (2)
C20	0.024 (2)	0.023 (2)	0.037 (3)	0.011 (2)	0.003 (2)	0.002 (2)
C21	0.020 (2)	0.015 (2)	0.031 (2)	0.0096 (18)	0.0005 (19)	0.0052 (18)
C22	0.026 (2)	0.025 (2)	0.030 (2)	0.012 (2)	0.005 (2)	0.005 (2)
C23	0.018 (2)	0.033 (3)	0.046 (3)	0.007 (2)	0.006 (2)	0.007 (2)
C24	0.023 (3)	0.030 (3)	0.050 (3)	0.012 (2)	−0.010 (2)	−0.002 (2)
C25	0.030 (3)	0.025 (3)	0.036 (3)	0.010 (2)	−0.005 (2)	0.002 (2)
C26	0.025 (2)	0.025 (2)	0.036 (3)	0.012 (2)	0.008 (2)	0.010 (2)
C27	0.022 (2)	0.033 (3)	0.019 (2)	0.011 (2)	−0.0023 (19)	0.003 (2)
C28	0.034 (3)	0.029 (3)	0.023 (2)	0.014 (2)	0.000 (2)	0.001 (2)
C29	0.036 (3)	0.040 (3)	0.021 (2)	0.010 (2)	−0.007 (2)	−0.001 (2)
C30	0.027 (3)	0.043 (3)	0.030 (3)	0.006 (2)	−0.006 (2)	0.006 (2)
C31	0.033 (3)	0.051 (3)	0.039 (3)	0.025 (3)	0.000 (2)	0.007 (3)
C32	0.035 (3)	0.032 (3)	0.028 (2)	0.015 (2)	−0.003 (2)	0.001 (2)
C33	0.039 (3)	0.041 (3)	0.020 (2)	0.022 (3)	−0.003 (2)	−0.004 (2)
C34	0.053 (4)	0.045 (3)	0.033 (3)	0.021 (3)	−0.008 (3)	0.005 (3)
C35	0.055 (4)	0.044 (3)	0.043 (3)	0.015 (3)	−0.014 (3)	−0.005 (3)
C36	0.061 (4)	0.042 (3)	0.029 (3)	0.021 (3)	0.004 (3)	0.002 (2)
C37	0.054 (4)	0.044 (3)	0.030 (3)	0.032 (3)	0.000 (2)	0.002 (2)
C38	0.047 (3)	0.048 (3)	0.022 (2)	0.027 (3)	0.000 (2)	−0.003 (2)
C39	0.044 (4)	0.088 (6)	0.051 (4)	0.008 (4)	−0.017 (3)	0.001 (4)
C40	0.052 (4)	0.058 (4)	0.041 (3)	−0.011 (3)	−0.017 (3)	0.001 (3)
C41	0.056 (5)	0.159 (9)	0.069 (5)	−0.018 (6)	−0.032 (4)	0.040 (6)
C42	0.065 (5)	0.139 (8)	0.044 (4)	0.039 (5)	0.004 (4)	0.005 (5)
C43	0.069 (5)	0.114 (7)	0.058 (4)	0.047 (5)	−0.016 (4)	−0.020 (5)
C44	0.054 (4)	0.115 (7)	0.062 (4)	0.044 (5)	−0.022 (4)	−0.036 (4)
C45	0.039 (3)	0.065 (4)	0.061 (4)	0.013 (3)	−0.012 (3)	−0.008 (3)
C46	0.055 (4)	0.086 (5)	0.042 (3)	0.048 (4)	−0.019 (3)	−0.021 (3)
C47	0.070 (5)	0.123 (7)	0.079 (5)	0.051 (5)	−0.026 (4)	−0.033 (5)
C48	0.070 (5)	0.075 (5)	0.107 (7)	0.035 (4)	−0.019 (5)	−0.015 (5)
C49	0.066 (5)	0.073 (5)	0.073 (5)	0.034 (4)	−0.024 (4)	−0.015 (4)
C50	0.047 (4)	0.050 (4)	0.103 (6)	0.020 (3)	−0.035 (4)	−0.009 (4)
C51	0.053 (4)	0.060 (4)	0.082 (5)	0.023 (4)	−0.023 (4)	−0.006 (4)
C52	0.032 (3)	0.054 (4)	0.060 (4)	0.018 (3)	−0.005 (3)	0.002 (3)
C53	0.063 (5)	0.058 (5)	0.176 (10)	0.019 (4)	−0.042 (6)	0.008 (5)
C54	0.065 (5)	0.063 (5)	0.102 (6)	0.021 (4)	−0.020 (5)	0.008 (4)
C55	0.055 (4)	0.081 (6)	0.071 (5)	0.023 (4)	−0.004 (4)	0.004 (4)
C56	0.042 (4)	0.074 (5)	0.049 (4)	0.003 (3)	−0.009 (3)	0.015 (3)
O1	0.057 (2)	0.059 (3)	0.042 (2)	0.042 (2)	−0.0079 (19)	−0.009 (2)
O2	0.101 (4)	0.089 (4)	0.070 (3)	0.073 (3)	−0.002 (3)	−0.014 (3)
P1	0.0213 (6)	0.0213 (6)	0.0294 (11)	0.0106 (3)	0.000	0.000
P2	0.0181 (6)	0.0159 (6)	0.0278 (6)	0.0068 (5)	0.0022 (5)	0.0028 (5)
P3	0.0246 (6)	0.0246 (6)	0.0199 (10)	0.0123 (3)	0.000	0.000
P4	0.0368 (8)	0.0368 (8)	0.0208 (10)	0.0184 (4)	0.000	0.000
P5	0.0368 (8)	0.0461 (8)	0.0302 (7)	0.0171 (7)	−0.0067 (6)	−0.0058 (6)
Ag1	0.0379 (3)	0.0379 (3)	0.0295 (3)	0.01896 (13)	0.000	0.000
Ag2	0.0183 (2)	0.0183 (2)	0.0213 (3)	0.00914 (10)	0.000	0.000
Ag3	0.0367 (3)	0.0367 (3)	0.0239 (3)	0.01836 (13)	0.000	0.000
N1	0.029 (2)	0.029 (2)	0.034 (4)	0.0144 (11)	0.000	0.000

### Geometric parameters (Å, º)

**Table:**

C1—N1	1.460 (6)	C33—P4	1.841 (5)
C1—C2	1.541 (9)	C34—C35	1.399 (8)
C1—H1A	0.9700	C34—H34	0.9300
C1—H1B	0.9700	C35—C36	1.413 (8)
C2—O1	1.227 (7)	C35—H35	0.9300
C2—O2	1.263 (7)	C36—C37	1.349 (8)
C3—C8	1.380 (6)	C36—H36	0.9300
C3—C4	1.395 (6)	C37—C38	1.402 (7)
C3—P1	1.821 (4)	C37—H37	0.9300
C4—C5	1.381 (7)	C38—H38	0.9300
C4—H4	0.9300	C39—C40	1.361 (9)
C5—C6	1.385 (7)	C39—C44	1.420 (10)
C5—H5	0.9300	C39—P5	1.826 (7)
C6—C7	1.384 (7)	C40—C41	1.381 (9)
C6—H6	0.9300	C40—H40	0.9300
C7—C8	1.376 (7)	C41—C42	1.419 (13)
C7—H7	0.9300	C41—H41	0.9300
C8—H8	0.9300	C42—C43	1.407 (11)
C9—C10	1.382 (6)	C42—H42	0.9300
C9—C14	1.393 (6)	C43—C44	1.397 (10)
C9—P2	1.839 (5)	C43—H43	0.9300
C10—C11	1.399 (6)	C44—H44	0.9300
C10—H10	0.9300	C45—C50	1.395 (9)
C11—C12	1.367 (7)	C45—C46	1.406 (8)
C11—H11	0.9300	C45—P5	1.830 (7)
C12—C13	1.396 (7)	C46—C47	1.388 (10)
C12—H12	0.9300	C46—H46	0.9300
C13—C14	1.378 (7)	C47—C48	1.417 (11)
C13—H13	0.9300	C47—H47	0.9300
C14—H14	0.9300	C48—C49	1.406 (10)
C15—C16	1.387 (6)	C48—H48	0.9300
C15—C20	1.403 (6)	C49—C50	1.404 (10)
C15—P2	1.824 (4)	C49—H49	0.9300
C16—C17	1.392 (6)	C50—H50	0.9300
C16—H16	0.9300	C51—C52	1.399 (9)
C17—C18	1.368 (7)	C51—C56	1.441 (10)
C17—H17	0.9300	C51—P5	1.778 (7)
C18—C19	1.379 (7)	C52—C53	1.310 (10)
C18—H18	0.9300	C52—H52	0.9300
C19—C20	1.397 (7)	C53—C54	1.464 (12)
C19—H19	0.9300	C53—H53	0.9300
C20—H20	0.9300	C54—C55	1.388 (11)
C21—C26	1.387 (6)	C54—H54	0.9300
C21—C22	1.394 (6)	C55—C56	1.399 (10)
C21—P2	1.828 (4)	C55—H55	0.9300
C22—C23	1.403 (6)	C56—H56	0.9300
C22—H22	0.9300	O1—Ag1	2.599 (4)
C23—C24	1.383 (7)	P1—C3^i^	1.821 (4)
C23—H23	0.9300	P1—C3^ii^	1.821 (5)
C24—C25	1.392 (7)	P1—Ag1	2.339 (2)
C24—H24	0.9300	P2—Ag2	2.6210 (11)
C25—C26	1.370 (6)	P3—C27^iii^	1.838 (5)
C25—H25	0.9300	P3—C27^iv^	1.838 (4)
C26—H26	0.9300	P3—Ag2	2.589 (2)
C27—C28	1.384 (6)	P4—C33^iii^	1.841 (5)
C27—C32	1.397 (7)	P4—C33^iv^	1.841 (5)
C27—P3	1.838 (4)	P4—Ag3	2.582 (2)
C28—C29	1.391 (7)	P5—Ag3	2.5862 (14)
C28—H28	0.9300	Ag1—N1	2.324 (7)
C29—C30	1.382 (8)	Ag1—O1^ii^	2.599 (4)
C29—H29	0.9300	Ag1—O1^i^	2.599 (4)
C30—C31	1.385 (7)	Ag2—P2^iii^	2.6210 (11)
C30—H30	0.9300	Ag2—P2^iv^	2.6211 (11)
C31—C32	1.378 (7)	Ag3—P5^iii^	2.5861 (14)
C31—H31	0.9300	Ag3—P5^iv^	2.5861 (14)
C32—H32	0.9300	N1—C1^i^	1.460 (6)
C33—C38	1.379 (7)	N1—C1^ii^	1.460 (6)
C33—C34	1.384 (7)		
			
N1—C1—C2	113.5 (5)	C33—C38—H38	120.0
N1—C1—H1A	108.9	C37—C38—H38	120.0
C2—C1—H1A	108.9	C40—C39—C44	120.4 (6)
N1—C1—H1B	108.9	C40—C39—P5	119.8 (5)
C2—C1—H1B	108.9	C44—C39—P5	119.9 (6)
H1A—C1—H1B	107.7	C39—C40—C41	123.7 (7)
O1—C2—O2	125.7 (6)	C39—C40—H40	118.1
O1—C2—C1	119.6 (5)	C41—C40—H40	118.1
O2—C2—C1	114.8 (6)	C40—C41—C42	117.0 (8)
C8—C3—C4	118.7 (4)	C40—C41—H41	121.5
C8—C3—P1	118.4 (3)	C42—C41—H41	121.5
C4—C3—P1	122.9 (3)	C43—C42—C41	119.7 (7)
C5—C4—C3	120.1 (5)	C43—C42—H42	120.1
C5—C4—H4	119.9	C41—C42—H42	120.1
C3—C4—H4	119.9	C44—C43—C42	121.7 (8)
C4—C5—C6	120.3 (5)	C44—C43—H43	119.2
C4—C5—H5	119.9	C42—C43—H43	119.2
C6—C5—H5	119.9	C43—C44—C39	117.2 (8)
C7—C6—C5	119.8 (5)	C43—C44—H44	121.4
C7—C6—H6	120.1	C39—C44—H44	121.4
C5—C6—H6	120.1	C50—C45—C46	123.0 (7)
C8—C7—C6	119.6 (5)	C50—C45—P5	120.4 (6)
C8—C7—H7	120.2	C46—C45—P5	116.6 (5)
C6—C7—H7	120.2	C47—C46—C45	118.8 (6)
C7—C8—C3	121.5 (5)	C47—C46—H46	120.6
C7—C8—H8	119.2	C45—C46—H46	120.6
C3—C8—H8	119.2	C46—C47—C48	120.3 (7)
C10—C9—C14	119.0 (4)	C46—C47—H47	119.9
C10—C9—P2	123.2 (3)	C48—C47—H47	119.9
C14—C9—P2	117.8 (3)	C49—C48—C47	119.0 (8)
C9—C10—C11	120.7 (4)	C49—C48—H48	120.5
C9—C10—H10	119.7	C47—C48—H48	120.5
C11—C10—H10	119.7	C50—C49—C48	121.9 (7)
C12—C11—C10	119.7 (4)	C50—C49—H49	119.0
C12—C11—H11	120.1	C48—C49—H49	119.0
C10—C11—H11	120.1	C45—C50—C49	116.9 (7)
C11—C12—C13	120.2 (5)	C45—C50—H50	121.5
C11—C12—H12	119.9	C49—C50—H50	121.5
C13—C12—H12	119.9	C52—C51—C56	118.8 (7)
C14—C13—C12	120.0 (5)	C52—C51—P5	121.3 (6)
C14—C13—H13	120.0	C56—C51—P5	119.9 (6)
C12—C13—H13	120.0	C53—C52—C51	125.9 (7)
C13—C14—C9	120.5 (4)	C53—C52—H52	117.1
C13—C14—H14	119.8	C51—C52—H52	117.1
C9—C14—H14	119.8	C52—C53—C54	116.9 (8)
C16—C15—C20	119.0 (4)	C52—C53—H53	121.6
C16—C15—P2	119.4 (3)	C54—C53—H53	121.6
C20—C15—P2	121.7 (3)	C55—C54—C53	118.6 (8)
C15—C16—C17	120.4 (4)	C55—C54—H54	120.7
C15—C16—H16	119.8	C53—C54—H54	120.7
C17—C16—H16	119.8	C54—C55—C56	123.6 (8)
C18—C17—C16	120.7 (4)	C54—C55—H55	118.2
C18—C17—H17	119.7	C56—C55—H55	118.2
C16—C17—H17	119.7	C55—C56—C51	115.8 (8)
C17—C18—C19	119.7 (5)	C55—C56—H56	122.1
C17—C18—H18	120.1	C51—C56—H56	122.1
C19—C18—H18	120.1	C2—O1—Ag1	108.3 (4)
C18—C19—C20	120.8 (5)	C3^i^—P1—C3^ii^	104.14 (18)
C18—C19—H19	119.6	C3^i^—P1—C3	104.14 (18)
C20—C19—H19	119.6	C3^ii^—P1—C3	104.14 (18)
C19—C20—C15	119.5 (4)	C3^i^—P1—Ag1	114.39 (15)
C19—C20—H20	120.3	C3^ii^—P1—Ag1	114.39 (15)
C15—C20—H20	120.3	C3—P1—Ag1	114.39 (15)
C26—C21—C22	118.7 (4)	C15—P2—C21	101.7 (2)
C26—C21—P2	118.1 (3)	C15—P2—C9	103.28 (19)
C22—C21—P2	123.2 (3)	C21—P2—C9	102.69 (19)
C21—C22—C23	120.1 (4)	C15—P2—Ag2	116.05 (14)
C21—C22—H22	120.0	C21—P2—Ag2	116.10 (14)
C23—C22—H22	120.0	C9—P2—Ag2	115.02 (14)
C24—C23—C22	119.8 (4)	C27^iii^—P3—C27^iv^	102.95 (17)
C24—C23—H23	120.1	C27^iii^—P3—C27	102.95 (17)
C22—C23—H23	120.1	C27^iv^—P3—C27	102.95 (17)
C23—C24—C25	120.0 (4)	C27^iii^—P3—Ag2	115.39 (15)
C23—C24—H24	120.0	C27^iv^—P3—Ag2	115.39 (15)
C25—C24—H24	120.0	C27—P3—Ag2	115.40 (15)
C26—C25—C24	119.8 (5)	C33—P4—C33^iii^	102.83 (18)
C26—C25—H25	120.1	C33—P4—C33^iv^	102.83 (18)
C24—C25—H25	120.1	C33^iii^—P4—C33^iv^	102.83 (18)
C25—C26—C21	121.6 (4)	C33—P4—Ag3	115.49 (15)
C25—C26—H26	119.2	C33^iii^—P4—Ag3	115.50 (15)
C21—C26—H26	119.2	C33^iv^—P4—Ag3	115.50 (15)
C28—C27—C32	118.8 (4)	C51—P5—C39	104.4 (3)
C28—C27—P3	123.3 (4)	C51—P5—C45	105.6 (3)
C32—C27—P3	117.9 (3)	C39—P5—C45	103.1 (3)
C27—C28—C29	120.2 (5)	C51—P5—Ag3	114.1 (2)
C27—C28—H28	119.9	C39—P5—Ag3	114.4 (2)
C29—C28—H28	119.9	C45—P5—Ag3	114.0 (2)
C30—C29—C28	120.0 (5)	N1—Ag1—P1	180.0
C30—C29—H29	120.0	N1—Ag1—O1^ii^	67.92 (8)
C28—C29—H29	120.0	P1—Ag1—O1^ii^	112.08 (8)
C29—C30—C31	120.4 (5)	N1—Ag1—O1^i^	67.92 (8)
C29—C30—H30	119.8	P1—Ag1—O1^i^	112.08 (8)
C31—C30—H30	119.8	O1^ii^—Ag1—O1^i^	106.74 (9)
C32—C31—C30	119.2 (5)	N1—Ag1—O1	67.92 (8)
C32—C31—H31	120.4	P1—Ag1—O1	112.08 (8)
C30—C31—H31	120.4	O1^ii^—Ag1—O1	106.74 (9)
C31—C32—C27	121.3 (5)	O1^i^—Ag1—O1	106.74 (9)
C31—C32—H32	119.4	P3—Ag2—P2^iii^	109.19 (3)
C27—C32—H32	119.4	P3—Ag2—P2	109.19 (3)
C38—C33—C34	119.7 (5)	P2^iii^—Ag2—P2	109.75 (3)
C38—C33—P4	123.2 (4)	P3—Ag2—P2^iv^	109.19 (3)
C34—C33—P4	117.1 (4)	P2^iii^—Ag2—P2^iv^	109.75 (3)
C33—C34—C35	120.1 (5)	P2—Ag2—P2^iv^	109.75 (3)
C33—C34—H34	120.0	P4—Ag3—P5^iii^	110.11 (3)
C35—C34—H34	120.0	P4—Ag3—P5^iv^	110.11 (3)
C34—C35—C36	119.7 (6)	P5^iii^—Ag3—P5^iv^	108.83 (3)
C34—C35—H35	120.1	P4—Ag3—P5	110.11 (3)
C36—C35—H35	120.1	P5^iii^—Ag3—P5	108.83 (3)
C37—C36—C35	119.2 (5)	P5^iv^—Ag3—P5	108.82 (3)
C37—C36—H36	120.4	C1—N1—C1^i^	111.5 (3)
C35—C36—H36	120.4	C1—N1—C1^ii^	111.5 (3)
C36—C37—C38	121.3 (5)	C1^i^—N1—C1^ii^	111.5 (3)
C36—C37—H37	119.3	C1—N1—Ag1	107.4 (3)
C38—C37—H37	119.3	C1^i^—N1—Ag1	107.4 (3)
C33—C38—C37	120.0 (5)	C1^ii^—N1—Ag1	107.4 (3)
			
N1—C1—C2—O1	−15.2 (8)	P5—C51—C52—C53	176.6 (7)
N1—C1—C2—O2	165.8 (5)	C51—C52—C53—C54	6.8 (12)
C8—C3—C4—C5	0.7 (7)	C52—C53—C54—C55	−7.4 (12)
P1—C3—C4—C5	−178.0 (4)	C53—C54—C55—C56	3.4 (12)
C3—C4—C5—C6	0.8 (7)	C54—C55—C56—C51	1.5 (10)
C4—C5—C6—C7	−2.0 (8)	C52—C51—C56—C55	−2.6 (9)
C5—C6—C7—C8	1.5 (8)	P5—C51—C56—C55	178.9 (5)
C6—C7—C8—C3	0.0 (8)	O2—C2—O1—Ag1	158.1 (6)
C4—C3—C8—C7	−1.1 (7)	C1—C2—O1—Ag1	−20.8 (7)
P1—C3—C8—C7	177.6 (4)	C8—C3—P1—C3^i^	−88.0 (5)
C14—C9—C10—C11	0.0 (7)	C4—C3—P1—C3^i^	90.7 (3)
P2—C9—C10—C11	−179.7 (3)	C8—C3—P1—C3^ii^	163.2 (4)
C9—C10—C11—C12	−0.8 (7)	C4—C3—P1—C3^ii^	−18.1 (4)
C10—C11—C12—C13	1.3 (7)	C8—C3—P1—Ag1	37.6 (4)
C11—C12—C13—C14	−1.0 (8)	C4—C3—P1—Ag1	−143.7 (3)
C12—C13—C14—C9	0.1 (7)	C16—C15—P2—C21	−154.3 (4)
C10—C9—C14—C13	0.3 (7)	C20—C15—P2—C21	25.4 (4)
P2—C9—C14—C13	−179.9 (4)	C16—C15—P2—C9	99.5 (4)
C20—C15—C16—C17	−0.1 (7)	C20—C15—P2—C9	−80.9 (4)
P2—C15—C16—C17	179.5 (3)	C16—C15—P2—Ag2	−27.3 (4)
C15—C16—C17—C18	0.7 (7)	C20—C15—P2—Ag2	152.3 (3)
C16—C17—C18—C19	−0.9 (7)	C26—C21—P2—C15	67.2 (4)
C17—C18—C19—C20	0.6 (8)	C22—C21—P2—C15	−113.8 (4)
C18—C19—C20—C15	−0.1 (7)	C26—C21—P2—C9	173.8 (3)
C16—C15—C20—C19	−0.2 (7)	C22—C21—P2—C9	−7.1 (4)
P2—C15—C20—C19	−179.8 (4)	C26—C21—P2—Ag2	−59.8 (4)
C26—C21—C22—C23	−0.7 (6)	C22—C21—P2—Ag2	119.3 (3)
P2—C21—C22—C23	−179.8 (3)	C10—C9—P2—C15	11.9 (4)
C21—C22—C23—C24	−0.6 (7)	C14—C9—P2—C15	−167.9 (3)
C22—C23—C24—C25	0.7 (7)	C10—C9—P2—C21	−93.6 (4)
C23—C24—C25—C26	0.6 (7)	C14—C9—P2—C21	86.6 (4)
C24—C25—C26—C21	−2.0 (7)	C10—C9—P2—Ag2	139.3 (3)
C22—C21—C26—C25	2.1 (6)	C14—C9—P2—Ag2	−40.4 (4)
P2—C21—C26—C25	−178.8 (4)	C28—C27—P3—C27^iii^	−102.6 (3)
C32—C27—C28—C29	0.6 (7)	C32—C27—P3—C27^iii^	77.7 (5)
P3—C27—C28—C29	−179.1 (4)	C28—C27—P3—C27^iv^	4.2 (5)
C27—C28—C29—C30	−2.2 (7)	C32—C27—P3—C27^iv^	−175.5 (3)
C28—C29—C30—C31	2.2 (7)	C28—C27—P3—Ag2	130.8 (4)
C29—C30—C31—C32	−0.7 (8)	C32—C27—P3—Ag2	−48.9 (4)
C30—C31—C32—C27	−0.8 (7)	C38—C33—P4—C33^iii^	101.8 (3)
C28—C27—C32—C31	0.9 (7)	C34—C33—P4—C33^iii^	−78.0 (5)
P3—C27—C32—C31	−179.4 (4)	C38—C33—P4—C33^iv^	−4.8 (5)
C38—C33—C34—C35	0.9 (8)	C34—C33—P4—C33^iv^	175.4 (4)
P4—C33—C34—C35	−179.3 (4)	C38—C33—P4—Ag3	−131.5 (4)
C33—C34—C35—C36	0.5 (9)	C34—C33—P4—Ag3	48.7 (4)
C34—C35—C36—C37	−1.2 (9)	C52—C51—P5—C39	−72.5 (6)
C35—C36—C37—C38	0.6 (8)	C56—C51—P5—C39	105.9 (6)
C34—C33—C38—C37	−1.5 (7)	C52—C51—P5—C45	179.2 (5)
P4—C33—C38—C37	178.6 (4)	C56—C51—P5—C45	−2.4 (6)
C36—C37—C38—C33	0.8 (8)	C52—C51—P5—Ag3	53.2 (6)
C44—C39—C40—C41	−1.4 (13)	C56—C51—P5—Ag3	−128.4 (5)
P5—C39—C40—C41	179.2 (7)	C40—C39—P5—C51	175.4 (7)
C39—C40—C41—C42	4.7 (14)	C44—C39—P5—C51	−4.0 (7)
C40—C41—C42—C43	−6.2 (14)	C40—C39—P5—C45	−74.4 (7)
C41—C42—C43—C44	4.8 (13)	C44—C39—P5—C45	106.2 (6)
C42—C43—C44—C39	−1.4 (11)	C40—C39—P5—Ag3	49.9 (7)
C40—C39—C44—C43	−0.4 (11)	C44—C39—P5—Ag3	−129.5 (5)
P5—C39—C44—C43	179.0 (5)	C50—C45—P5—C51	101.5 (6)
C50—C45—C46—C47	3.9 (11)	C46—C45—P5—C51	−76.4 (6)
P5—C45—C46—C47	−178.2 (6)	C50—C45—P5—C39	−7.8 (6)
C45—C46—C47—C48	−4.1 (12)	C46—C45—P5—C39	174.3 (5)
C46—C47—C48—C49	2.7 (13)	C50—C45—P5—Ag3	−132.4 (5)
C47—C48—C49—C50	−1.0 (12)	C46—C45—P5—Ag3	49.7 (6)
C46—C45—C50—C49	−2.1 (10)	C2—C1—N1—C1^i^	163.9 (5)
P5—C45—C50—C49	−179.9 (5)	C2—C1—N1—C1^ii^	−70.9 (8)
C48—C49—C50—C45	0.6 (11)	C2—C1—N1—Ag1	46.5 (5)
C56—C51—C52—C53	−1.8 (11)		

Symmetry codes: (i) −*x*+*y*+1, −*x*+2, *z*; (ii) −*y*+2, *x*−*y*+1, *z*; (iii) −*x*+*y*+1, −*x*+1, *z*; (iv) −*y*+1, *x*−*y*, *z*.
